# Dual T-cell constant β chain (TRBC)1 and TRBC2 staining for the identification of T-cell neoplasms by flow cytometry

**DOI:** 10.1038/s41408-024-01002-0

**Published:** 2024-02-29

**Authors:** Pedro Horna, Matthew J. Weybright, Mathieu Ferrari, Dennis Jungherz, YaYi Peng, Zulaikha Akbar, F. Tudor Ilca, Gregory E. Otteson, Jansen N. Seheult, Janosch Ortmann, Min Shi, Paul M. Maciocia, Marco Herling, Martin A. Pule, Horatiu Olteanu

**Affiliations:** 1 Division of Hematopathology, Mayo Clinic, Rochester, MN USA; 2 Autolus Ltd, London, UK; 3 Department of Internal Medicine, University of Cologne, Cologne, Germany; 4 Centre de Recherches Mathematiques, Universite du Quebec a Montreal, Montreal, Canada; 5 Cancer Institute, University College London, London, UK

**Keywords:** T-cell lymphoma, T-cell lymphoma

## Abstract

The diagnosis of leukemic T-cell malignancies is often challenging, due to overlapping features with reactive T-cells and limitations of currently available T-cell clonality assays. Recently developed therapeutic antibodies specific for the mutually exclusive T-cell receptor constant β chain (TRBC)1 and TRBC2 isoforms provide a unique opportunity to assess for TRBC-restriction as a surrogate of clonality in the flow cytometric analysis of T-cell neoplasms. To demonstrate the diagnostic utility of this approach, we studied 164 clinical specimens with (60) or without (104) T-cell neoplasia, in addition to 39 blood samples from healthy donors. Dual TRBC1 and TRBC2 expression was studied within a comprehensive T-cell panel, in a fashion similar to the routine evaluation of kappa and lambda immunoglobulin light chains for the detection of clonal B-cells. Polytypic TRBC expression was demonstrated on total, CD4^+^ and CD8^+^ T-cells from all healthy donors; and by intracellular staining on benign T-cell precursors. All neoplastic T-cells were TRBC-restricted, except for 8 cases (13%) lacking TRBC expression. T-cell clones of uncertain significance were identified in 17 samples without T-cell malignancy (13%) and accounted for smaller subsets than neoplastic clones (median: 4.7 vs. 69% of lymphocytes, *p* < 0.0001). Single staining for TRBC1 produced spurious TRBC1-dim subsets in 24 clinical specimens (15%), all of which resolved with dual TRBC1/2 staining. Assessment of TRBC restriction by flow cytometry provides a rapid diagnostic method to detect clonal T-cells, and to accurately determine the targetable TRBC isoform expressed by T-cell malignancies.

## Introduction

The laboratory diagnosis of leukemic T-cell lymphoproliferative disorders neoplasms relies on the identification of cytologically and/or immunophenotypically abnormal T-cell populations, often in correlation with clinical findings and ancillary laboratory testing [1]. However, confident interpretation of an atypical T-cell population as diagnostic for T-cell malignancy is often challenging, given that similar atypical T-cell subsets can occasionally be encountered in reactive settings. Moreover, some leukemic T-cell neoplasms may lack overt immunophenotypic aberrancies and/or convincing cytologic abnormalities, precluding an unequivocal laboratory diagnosis [2, 3].

The diagnosis of B-cell neoplasms has been largely facilitated by the broad utilization of stains for kappa and lambda immunoglobulin light chains to assess for immune receptor monotypia indicative of B-cell clonality [4]. Such a simplified and effective approach has not been available for the analysis of T-cells in routine clinical diagnostics. Instead, detection of clonal T-cell receptor (TCR) gene rearrangements by multiplex polymerase chain reaction is commonly utilized [5]. This is a qualitative approach that lacks immunophenotypic information, is subject to interpretative challenges [6], and can produce false positive results in the setting of aging and inflammation [7, 8]. Alternatively, analysis of the TCR variable β repertoire (TCR-Vβ) by flow cytometry can be used [9, 10], but this method is labor-intensive, costly, often difficult to interpret and of limited sensitivity [11].

The gene encoding the TCR constant β chain (TRBC) has two isoforms: TRBC1 and TRBC2. In a manner analogous to light chain restriction, TCR gene rearrangement results in a TCR with the TCR β chain constant region encoded by either TRBC1 or TRBC2. This aspect of TCR gene-rearrangement has been described decades ago but is often overlooked, likely because unlike κ and λ light chains, TRBC1/2 encode almost identical proteins. We previously demonstrated that the anti-TCR antibody (JOVI.1) [12] has exquisite sensitivity for TRBC1, and this selectivity can be exploited to develop novel CAR T-cell therapies to deplete TRBC1^+^ malignant and benign T-cells while preserving T-cell immunity maintained by the TRBC2^+^ immune repertoire [13]. More recently, we employed computational biology and protein engineering to rationally design and produce mutant versions of JOVI.1 with switched specificity for TRBC2 and pre-clinical activity as CAR T-cell constructs targeting TRBC2^+^ T-cell malignancies [14].

The availability of complementary antibodies against TRBC1 and TRBC2 provides a unique opportunity to easily demonstrate T-cell clonality, in a fashion similar to detecting clonal B-cells based on kappa or lambda immunoglobulin light chain restriction. We hereby demonstrate the optimal performance of this approach for the confident laboratory diagnosis of T-cell neoplasms. In addition, we show that dual assessment of TRBC1/TRBC2 expression eliminates spurious TRBC-dim subsets on previously described TRBC1-only staining approaches, allowing for the accurate determination of the targetable TRBC isoform expressed by T-cell malignancies.

## Materials and methods

### Anti-TRBC2 antibody development

**Fig. 1:**
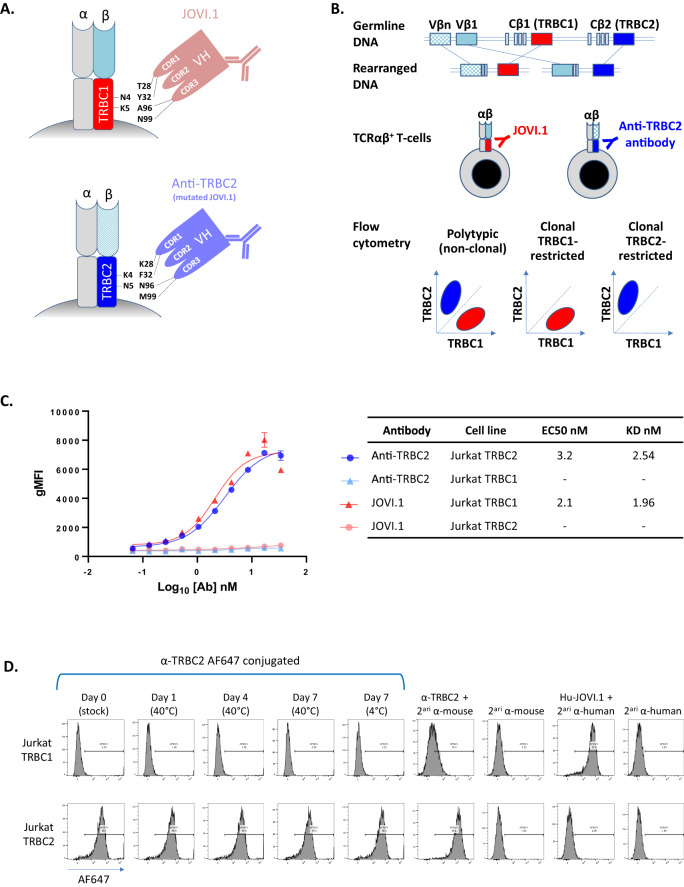
Strategic mutations on complementarity determining regions (CDR) of the JOVI.1 antibody result in switched specificity from T-cell receptor constant β chain (TRBC)1 to TRBC2, allowing for dual TRBC staining by flow cytometry. **A** Simplified model of JOVI.1 antibody binding to TRBC1 (top), showing key amino acid residues on both molecules responsible for the discriminative binding to one isoform only. Rationally-designed antibody mutations on CDR1 (T28K and Y32F) and CDR3 (A96N and N99M) of JOVI.1’s variable heavy chain (VH) domain results in switched specificity to TRBC2 (bottom). **B** T-cell receptor αβ gene rearrangement showing the random selection of 1 of 2 mutually exclusive TRBC genes. Anti-TRBC1 (JOVI.1) and anti-TRBC2 antibodies can be utilized in conjunction to assess for TRBC-restriction by flow cytometry as a surrogate for T-cell clonality. **C** Half maximal effective concentration (EC50) measurements and dissociation constant (KD) estimations (non-linear regression one site binding analysis) of anti-TRBC2 (blue and light blue) or JOVI.1 (red and pink) binding to TRBC1-positive (triangles) or TRBC2-positive (circles) Jurkat cells; as assessed by geometric mean fluorescence intensity (gMFI) using an anti-mouse IgG fluorescent-labeled secondary antibody. **D** Flow cytometry histograms showing the specificity of the anti-TRBC2 antibody for TRBC2-positive (top) as compared to TRBC1-positive (bottom) Jurkat cells; and stability of an Alexa Fluor(AF)-647-conjugated anti-TRBC2 antibody. Secondary (2^ari^) antibodies were AF-647 conjugates. A humanized JOVI.1 antibody (Hu-JOVI.1) is also shown as control.

A mouse anti-human TRBC2 antibody was specifically designed and developed for diagnostic flow cytometry, following a previously described strategy [14]. In short, an anti-TRBC2 antibody was produced based on structural engineering and rational design of mutations on complementarity determining region (CDR)1 (T28K and Y32F) and CDR3 (A96N and N99M) of the JOVI.1 antibody (Kabat numbering scheme), resulting in switched antibody specificity from TRBC1 to TRBC2 (Fig. 1A). The kinetic profile of the anti-TRBC2 antibody was studied against soluble TRBC1^+^ or TRBC2^+^ TCRs on a Biacore T200 surface plasmon resonance system (Cytiva, Marlborough, MA); and its thermal stability was assessed on a via Prometheus NT.48 nanoDSF differential scanning fluorimeter (NanoTemper, München, Germany) (Supplemental Fig. 1). Wild-type (WT) mycoplasma-free Jurkat cells (TRBC1^+^) and Jurkat cells engineered to express TRBC2 were evaluated by flow cytometry to confirm similar levels of surface CD3/TCR expression (Supplemental Fig. 2). These cell lines were then utilized to study the specificity and affinity of the anti-TRBC2 antibody compared to JOVI.1, using a secondary anti-mouse IgG (H + L) AF-647 antibody (Invitrogen, Waltham, MA), and staining with anti-CD3-PE-Cy7 (Biolegend, San Diego, CA) to gate on surface CD3/TCR-positive cells.

### Clinical specimens

As part of a test development effort, fresh clinical samples received for flow cytometric analysis at Mayo Clinic, Rochester, MN, were selected based on the presence or absence of involvement by a T-cell lymphoproliferative disorder, and availability of additional material remaining after diagnostic workup (Table 1). Peripheral blood specimens from healthy donors were obtained from Mayo Clinic’s biospecimen program. All test development data, diagnostic laboratory data, and corresponding medical records were retrospectively reviewed. This study was approved by the Mayo Clinic Institutional Review Board, including no requirement for patient consent based on minimal risk, adequate protection of patient’s confidentiality, and inability to obtain such consent retrospectively.

**Table 1:** Specimens and diagnoses.

Cohort	Diagnoses (*n*)	Specimens/patients	Specimen type (*n*)	Panels (**) (*n*)
**Healthy donors**	N/A	39 / 39	Peripheral blood (39)	T-cell (25) Sezary (21) Vβ/TRBC (5) Naïve/memory (10)
**Clinical specimens with no** **T-cell malignancy**	B-cell neoplasm (22) Inflammatory/autoimmune (25) Plasma cell proliferative disorder (13) Thymic epithelial neoplasia (9) Reactive cytopenia or cytosis (8) Carcinoma (8) Benign effusion (7) Myeloid neoplasm (7) Infectious (4) Benign thymic tissue (1)	104 / 102	Bone marrow (34) Lymph node / tissue (41) Peripheral blood (16) Body fluids (13)	T-cell (103) Sezary (11)
**T-cell neoplasms**	Cutaneous T-cell lymphoma (23) T lymphoblastic leukemia/lymphoma (11) T-cell large granular lymphocytic leukemia (9)(*) Peripheral T-cell lymphoma (12)(*) T-cell prolymphocytic leukemia (3) Lymphocytic variant hypereosinophilic syndrome (3) Hepatosplenic T-cell lymphoma (1)	60 / 57	Peripheral blood (38) Bone marrow (13) Lymph node / tissue (9)	T-cell (46) Sezary (18)

(*) Two samples from one patient had both peripheral T-cell lymphoma and T-cell large granular lymphocytic leukemia.

(**) Same samples were studied with more than one panel.

### Flow cytometry panels

Two comprehensive 11-color single-tube panels (“T-cell” and “Sezary” panels), including anti-TRBC1 and anti-TRBC2 antibodies, were developed at Mayo Clinic, Rochester, MN. A TCR-Vβ repertoire flow cytometry kit (IOTest Beta Mark, catalog number IM3497, Beckman Coulter, Brea, CA) was modified to assess for TRBC1 and TRBC2 expression in combination with TCR-Vβ classes on selected healthy donors (“Vβ/TRBC” panel). In addition, a “naïve/memory” panel was designed to study TRBC1 and TRBC2 expression on naive-memory CD4^+^ and CD8^+^ subsets defined based on CD62L and CD45RA expression [15, 16]. On all panels, our anti-TRBC2 antibody was pre-conjugated to Alexa Fluor 647. The T-cell, Sezary and naïve/memory panels were designed with a FITC-conjugated JOVI.1 (anti-TRBC1) antibody (Ancell Corporation, catalog number 101-040, Bayport, MN, USA), while the Vβ/TRBC panel included a Brilliant Violet 605-conjugated JOVI.1 antibody (BD Biosciences, catalog number 747979, Franklin Lakes, NJ) (Table 2). All surface staining steps were performed as previously described [17]. In cases where the cells of interest lacked surface expression of CD3 or TRBC, staining for cytoplasmic TRBC1 and TRBC2 (with or without cytoplasmic CD3) was performed after surface staining for all other antigens and standard fixation and permeabilization (FIX & PERM, Invitrogen, Waltham, MA). Events were acquired on a FACSLyric flow cytometer (BD Biosciences), with a target of 100,000 total cells. Routine diagnostic flow cytometry outside this test development activity was performed on clinical specimens using our TRBC1-only T-cell [17] and TRBC1-only Sezary [18] panels, as previously described.

**Table 2:** Flow cytometry panels.

Panel	Antigens tested	Fluorochromes
**T-cell**	CD2, CD3, CD4, CD5, CD7, CD8, CD19, CD45, TCRγδ, **TRBC1, TRBC2**	PerCP-Cy5.5, PE-Cy7, AF-700, BV-605, BV-421, APC-H7, BV-711, V500, PE, **FITC, AF-647**
**Sezary**	CD2, CD3, CD4, CD5, CD7, CD8, CD19, CD45, CD26, **TRBC1, TRBC2**	PerCP-Cy5.5, PE-Cy7, AF-700, BV-605, BV-421, APC-H7, BV-711, V500, PE, **FITC, AF-647**
**Vβ/TRBC**	Vβx, Vβy, CD3, **TRBC1, TRBC2** (x 8 tubes)	FITC, PE, PE-Cy7, **BV-605, AF-647**
**Naïve/** **memory**	CD3, CD4, CD8, CD45RA, CD62L, **TRBC1, TRBC2**	BV-605, AF-700, APC-H7, PE-Cy7, PE, **FITC, AF-647**

Antigens and fluorochromes are listed in corresponding order.

### Flow cytometry data analysis

Common normal T-cell subsets and immunophenotypically abnormal subsets consistent with a neoplastic T-cell population were manually gated by expert hematopathologists (PH and HO) on Kaluza version 2.1 (Beckman Coulter), based on patterns of expression of common surface T-cell antigens excluding TRBC1 and TRBC2. Expression of TRBC1 and TRBC2 on gated T-cell subsets of at least 200 events was evaluated on a TRBC1 vs. TRBC2 dot plot to assess for TRBC-restriction as surrogate for clonality (Fig. 1B). Thresholds for clonality were arbitrarily defined based on our extensive experience assessing TRBC1 expression only on clinical specimens (<15% or >85% TRBC1-positive events) [17–21], and applied on this study as percentage positivity (>85% TRBC1- or TRBC2-positive events) or equivalent TRBC2:TRBC1 ratios (TRBC2:TRBC1 <0.18 or >5.7). The development data using TRBC1/TRBC2 dual staining was compared to data collected during routine flow cytometric analysis using our previously validated TRBC1-only panels [17, 18].

### Statistical analysis

All statistic calculations were performed using GraphPad Prism, version 10.0.2 for Windows (GraphPad Software, San Diego, CA, USA). Comparisons of measurement values between two groups were performed using the Mann–Whitney test (clone size and TRBC2:TRBC1 ratios) or an unpaired two-tailed *t*- test (TCR-Vβ class percentages, age). A receiver operating characteristic (ROC) curve was constructed based on the maximum of %TRBC1^+^ and %TRBC2^+^ events for each gated TRBC-expressing tumor population (true positives); compared to a similar maximum for each gated CD4^+^/CD8^−^, CD8^+^/CD4^−^, CD4^+^/CD8^+^, and CD4^−^/CD8^−^ TCRαβ T-cell subset (>200 events) from samples without T-cell neoplasia (true negatives). A statistically significant *P* value was considered as less than 0.05.

## Results

### A novel anti-TRBC2 antibody combined with JOVI.1 demonstrates TRBC polytypia on benign blood and bone marrow T-cells by flow cytometry

We first tested the specificity of a newly developed and strategically mutated JOVI.1 antibody (Fig. 1A, B) with a kinetic profile favoring recognition of TRBC2 over TRBC1 (Supplemental Fig. 1A). By flow cytometry, this anti-TRBC2 antibody bound to the surface of a genetically engineered TRBC2-positive Jurkat cell line, but not to wild-type TRBC1-positive Jurkat cells exhibiting comparable levels of CD3/TCR expression (Fig. 1C and Supplemental Fig. 2). Moreover, the cell line-based binding affinity for TRBC2 was comparable to that of JOVI.1 for TRBC1, based on similar dissociation constants (KD of 2.54 nM vs. 1.96 nM, respectively). Labeling of anti-TRBC2 with AF-647 resulted in a thermally stable flow cytometry reagent (Supplemental Fig. 1B) with preserved specific staining of TRBC2^+^ Jurkat cells only (Fig. 1D).

**Fig. 2:**
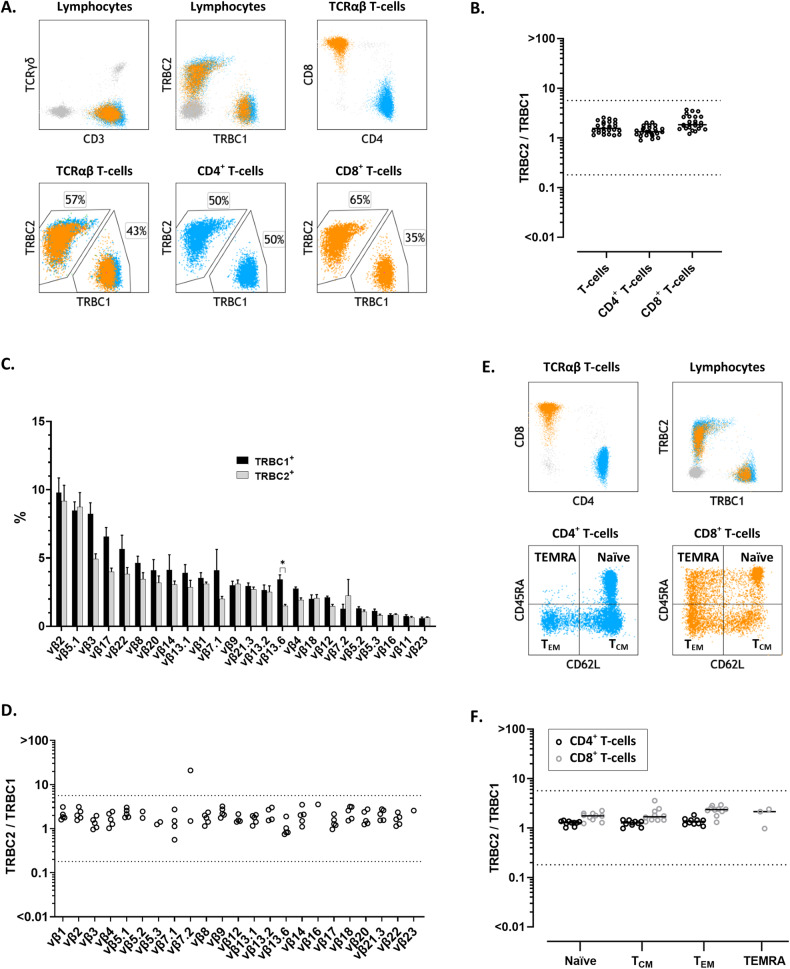
Dual staining for T-cell receptor constant β chain (TRBC)1 and TRBC2 demonstrates mutually exclusive and polytypic TRBC expression on benign T-cells and T-cell receptor variable β (TCR-Vβ) subsets. **A** Representative peripheral blood flow cytometry findings on a healthy donor showing polytypic TRBC expression on total, CD4^+^ (cyan) and CD8^+^ (orange) T-cells. **B** TRBC2:TRBC1 ratios of total, CD4^+^ and CD8^+^ T-cells from 25 healthy donor’s peripheral blood specimens. Solid lines: medians. Dotted lines: thresholds for clonality. **C** TCR-Vβ repertoire by flow cytometry on gated TRBC1^+^ (black bars) and TRBC1^-^ (gray bars) peripheral blood T-cells from five healthy donors, showing remarkably similar distributions. *p < 0.05. **D** TRBC2:TRBC1 ratios of each TCR-Vβ-positive T-cell subset from five healthy donors. Dotted lines depict thresholds for clonality. **E** Representative peripheral blood flow cytometry plots from a healthy donor showing distinct naïve, central memory (T_CM_), effector memory (T_EM_), and effector memory with reacquired CD45RA (T_EMRA_) subsets on the CD4^+^ (cyan) and CD8^+^ (orange) T-cell compartments. **F** TRBC2:TRBC1 ratios of gated naïve-memory CD4^+^ and CD8^+^ T-cell subsets from 10 healthy donors. Solid lines: medians. Dotted lines: thresholds for clonality.

We then studied peripheral blood specimens from 25 healthy donors, using a single-tube flow cytometry T-cell panel, including anti-TRBC2, JOVI.1 (anti-TRBC1), TCRγδ (to gate on TCRαβ by exclusion), and other antibodies recognizing common T-cell antigens (Tables 1,2). In all specimens, gated total TCRαβ T-cells, CD4^+^ T-cells, and CD8^+^ T-cells showed TRBC2:TRBC1 ratios consistent with polytypia, as evaluated using clonality thresholds based on our extensive experience using JOVI.1 staining only [17, 18, 20–22] (Fig. 2A, B). These clonality thresholds were further validated on receiver operating characteristic (ROC) curve analysis using tumor and control samples from this study (Supplemental Fig. 3). On healthy donors, the median TRBC2:TRBC1 ratios and interquartile ranges were 1.6 (1.3–2.0) for total TCRαβ T-cells, 1.3 (1.2–1.6) for CD4^+^ T-cells, and 1.8 (1.5–2.7) for TCRαβ CD8^+^ T-cells; with slightly higher TRBC2:TRBC1 ratios for CD8^+^ T-cells compared to CD4^+^ T-cells (*p* < 0.0001).

### TRBC1 and TRBC2 expression by flow cytometry is independent of TCR-Vβ restriction and naïve-memory immunophenotype

TCR-Vβ gene usage and TRBC gene selection are theoretically independent and probabilistically unrelated events during T-cell receptor gene rearrangement. This is a key assumption in the simplification of clonality assessment using TRBC1 and TRBC2 staining, as preferential TRBC usage associated with a subset of Vβ genes would produce false positive clonality results in settings where these Vβ subsets are enriched. To confirm this requirement, we compared the TCR-Vβ repertoire of gated TRBC1^+^ and TRBC2^+^ peripheral blood T-cells by flow cytometry on five healthy donors. As anticipated, both TCR-Vβ repertoires closely mirrored each other, with no discernable bias except for a slight but statistically significant skew of TCR-Vβ 13.6 for TRBC1 over TRBC2 (median: 3.4 vs. 1.5%; adjusted *p* = 0.01, Holm–Sidak method) (Fig. 2C). We also directly calculated the TRBC2:TRBC1 ratio of each TCR-Vβ subset on these five healthy donors. All detectable Vβ-positive subsets (≥200 cells) on all studied patients showed a polytypic TRBC2:TRBC1 ratio (Fig. 2D), except for a single donor harboring a small subset of TRBC2-restricted Vβ7.2^+^ T-cells (4.1% of lymphocytes) corresponding to a small CD8^+^ T-cell clone of uncertain significance (T-CUS) [17] on further evaluation (Supplemental Fig. 4). We then evaluated TRBC2:TRBC1 ratios on gated CD4^+^ and CD8^+^ naïve, central memory, effector memory and effector memory with reacquired CD45RA expression (TEMRA) subsets (Fig. 2E). As expected, all detectable effector-memory compartments (at least 200 events) from 10 healthy donors showed a polytypic TRBC2:TRBC1 ratio (Fig. 2F).

### Cytoplasmic TRBC1 and TRBC2 staining shows polytypic TRBC expression on benign maturing T-cell precursors

**Fig. 3:**
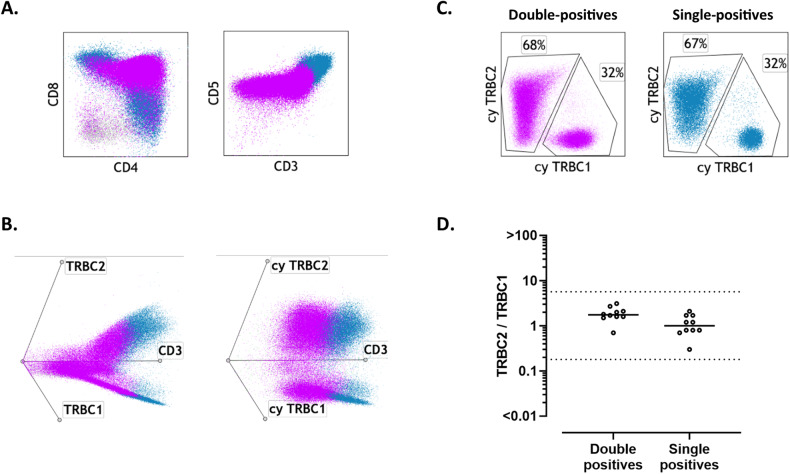
Benign T-cell precursors show polytypic T-cell receptor constant β chain (TRBC) expression using dual cytoplasmic TRBC1 and TRBC2 staining. **A** Representative immunophenotypic findings of a mediastinal mass involved by thymoma, showing benign CD4/CD8-double-positive T-cell precursors (violet) gradually acquiring surface CD3 expression as they mature into single-positive T-cells (blue). **B** Left: Double-positive T-cell precursors (violet) gradually acquire surface TRBC1 and TRBC2 expression while maturing into polytypic single-positive cells (blue). Right: Cytoplasmic TRBC1 and TRBC2 staining demonstrates polytypic TRBC expression on double-positive T-cell precursors. **C** Dual cytoplasmic TRBC1 and TRBC2 staining showing polytypic TRBC expression on double-positive and single-positive T-cell precursors. **D** TRBC2:TRBC1 ratios on double-positive and single-positive immature T-cells, using cytoplasmic dual staining on ten tissue biopsies with benign T-cell precursors.

As TCRβ gene rearrangement and protein expression are known to occur during early T-cell development [23], we hypothesized that cytoplasmic staining for TRBC1 and TRBC2 by flow cytometry should be able to demonstrate TRBC polytypia on benign immature T-cell precursors, despite the negative to dim surface TCR expression on most cells. To confirm this, we evaluated surface and cytoplasmic TRBC1 and TRBC2 expression (T-cell panel) on ten surgical biopsies harboring benign immature T-cell precursors (nine thymic epithelial tumors and one ectopic intrathyroidal thymic tissue). In all cases, CD4^+^/CD8^+^ double-positive and single-positive T-cell precursors showed detectable expression of cytoplasmic TRBC1 and TRBC2 by flow cytometry, with a polytypic TRBC2:TRBC1 ratio discernable throughout a broad maturation spectrum of progressive surface CD3/TCR expression (Fig. 3).

### TRBC-restriction is characteristic of T-cell neoplasms and small T-cell clones of uncertain significance

**Fig. 4:**
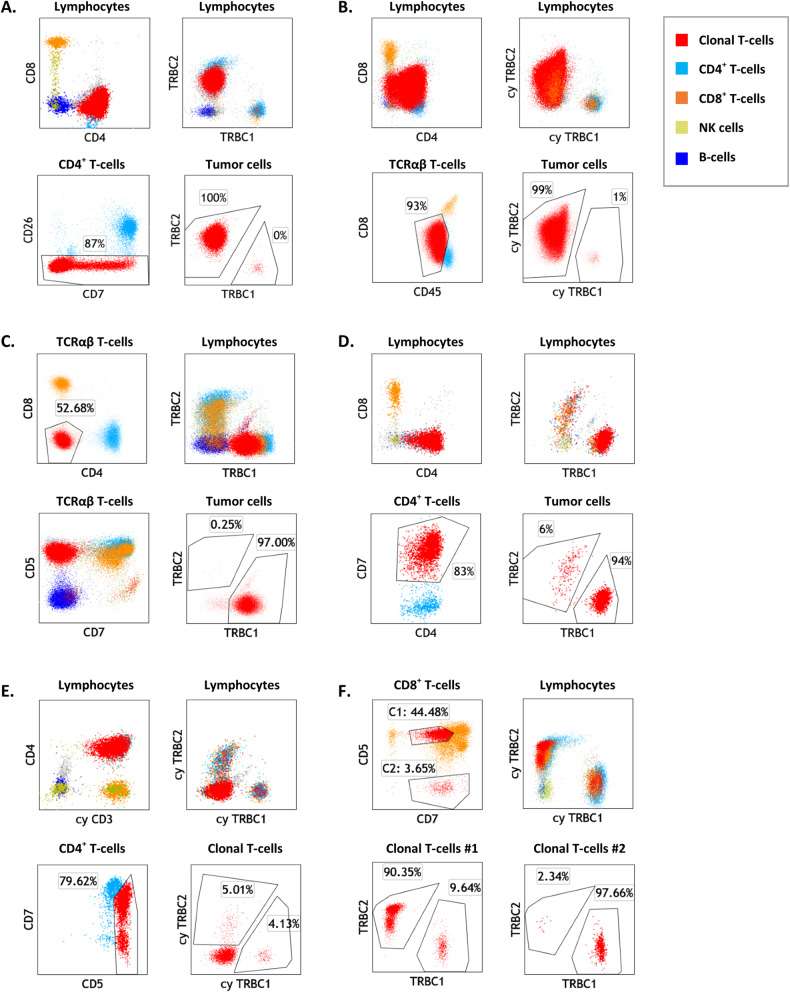
Dual T-cell receptor constant β chain (TRBC)1 and TRBC2 staining by flow cytometry demonstrates TRBC restriction on gated malignant T-cells. **A** Representative flow cytometry plots of peripheral blood involvement by Sezary syndrome (Sezary panel), showing a distinctly abnormal CD4^+^ T-cell subset (red) with loss of CD26 expression and TRBC2-restriction. Also shown are background polytypic CD4^+^ T-cells (cyan), CD8^+^ T-cells (orange), NK cells (gold) and B-cells (blue). **B** Peripheral blood involvement by T lymphoblastic leukemia/lymphoma (T-cell panel), showing an abnormal CD4-variable/CD8-dim T-cell population (red) that was surface CD3/TCR negative (data not shown) and on which TRBC2-restriction could be demonstrated by cytoplasmic (cy) TRBC1 and TRBC2 staining. **C** Inguinal lymph node involvement by cutaneous T-cell lymphoma, not otherwise specified, showing an expanded CD4/CD8-double-negative T-cell subset with TRBC1-restriction. **D** Cervical lymph node biopsy involved a CD4-positive peripheral T-cell lymphoma, showing a large subset of CD4^+^/CD7^+^ T-cells with TRBC1-restriction and absence of overt immunophenotypic aberrancies. **E** Peripheral blood from a patient with a lymphocytic variant of hypereosinophilic syndrome, showing an abnormal CD4^+^ T-cell subset negative for surface CD3 (not shown), positive for cytoplasmic CD3, and negative for surface and cytoplasmic TRBC. **F** Peripheral blood from a patient with Felty syndrome, showing 2 small CD8^+^ T-cell subsets with opposite TRBC-restriction, consistent with T-cell clones of uncertain significance.

**Fig. 5:**
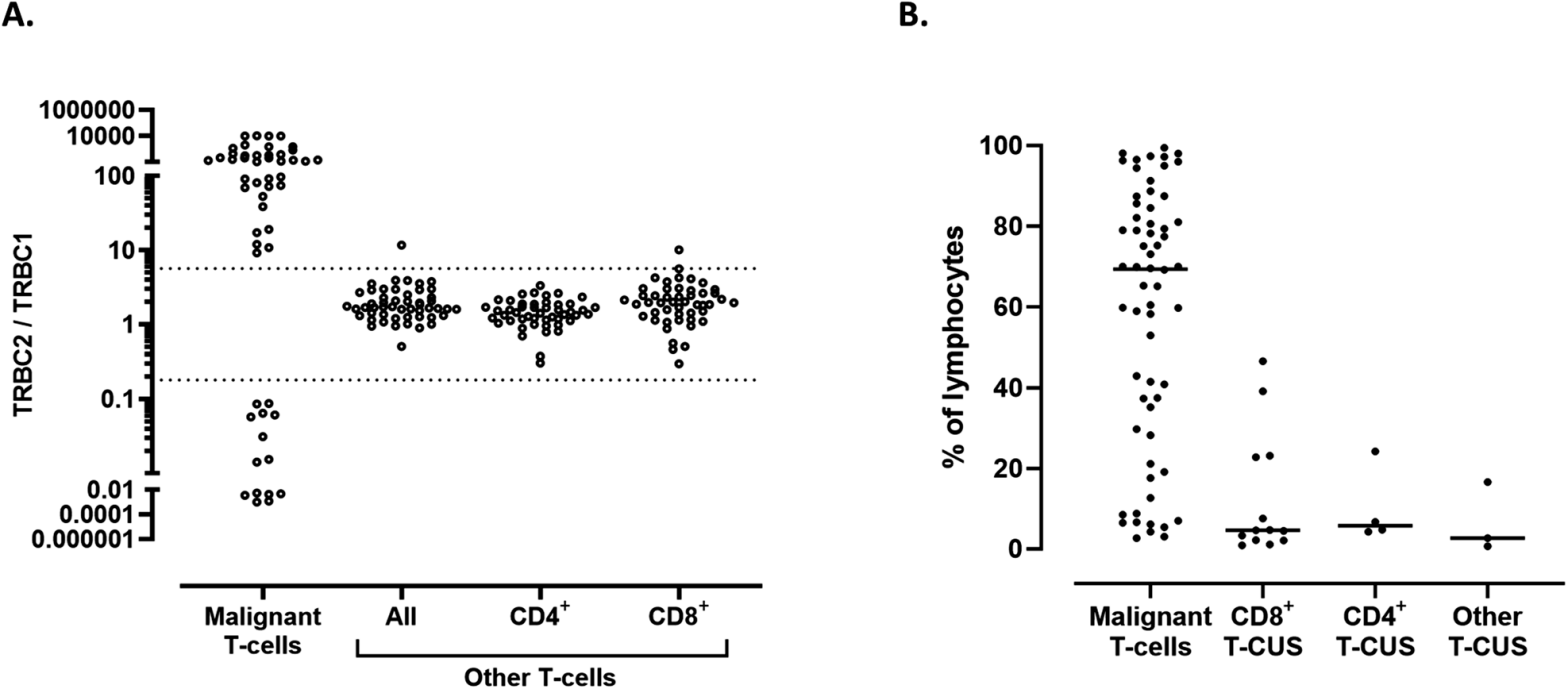
Evaluation of T-cell receptor constant β chain (TRBC)2:TRBC1 ratio on T-cell subsets identifies malignant populations and small T-cell clones of uncertain significance (T-CUS). **A** TRBC2:TRBC1 ratios of gated malignant T-cell populations from 52 clinical specimens with various T-cell neoplasms (excluding eight neoplasms lacking intracellular TRBC expression). Also shown are TRBC2:TRBC1 ratios of background (non-malignant) T-cells, including a blood sample with a small CD8^+^ T-cell clone of uncertain significance (outlier) in the setting of CD4^+^ cutaneous T-cell lymphoma. Dotted lines: thresholds for clonality. **B** Clone size of 62 T-cell neoplasms detected on 60 samples (expressed as a percentage of lymphocytes), compared to 20 T-CUS detected on 17 benign specimens.

We next evaluated 60 clinical specimens with confirmed involvement by various TCRαβ T-cell neoplasias, including cutaneous T-cell lymphoma (CTCL, 23), T lymphoblastic leukemia/lymphoma (T-LBL, 11), T-cell large granular lymphocytic leukemia (T-LGLL, 9), peripheral T-cell lymphoma (PTCL, 12), T-cell prolymphocytic leukemia (T-PLL, 3), a lymphocytic variant of hypereosinophilic syndrome (T-HES, 3), and hepatosplenic T-cell lymphoma (HSTCL, 1) (Tables 1,2). In 18 cases (30%, 9 T-LBL, 3 CTCL, 3 PTCL, 2 L-HES, and 1 HSTCL), cytoplasmic TRBC1 and TRBC2 staining was performed to assess for TRBC-restriction in the setting of dim to negative surface CD3 and/or TRBC expression. On all 60 cases involved by T-cell malignancy, expert gating showed a neoplastic subset exhibiting either a clonal TRBC2:TRBC1 ratio (52 cases, 87%)(Fig. 4A–D), or aberrant loss of both surface and cytoplasmic TRBC expression (8 cases, 13%: 4 T-ALL, 2 CTCL, 1 PTCL, and 1 L-HES) (Fig. 4E). Both TRBC1-restricted and TRBC2-restricted tumors were observed in all disease categories studied, with an overall higher incidence of TRBC2^+^ neoplasms (73%, 95% CI: 60–83%) as expected by the slight overrepresentation of TRBC2 in the normal T-cell repertoire. Moreover, all background total T-cells, CD4^+^ T-cells and CD8^+^ T-cells detectable (≥200 events) outside the expert-defined neoplastic gate showed a polytypic TRBC2:TRBC1 ratio; with the exception of a small CD8^+^ T-cell clone of uncertain significance (0.6% of lymphocytes) detected in the setting of blood involvement by CD4^+^ CTCL Fig. 5A). Overall our findings demonstrate that TRBC-restriction is a feature specific for the neoplastic T-cell subset.

Using a similar expert analysis, we evaluated 104 clinical specimens from patients with no T-cell malignancy and 29 blood specimens from healthy donors, for T-cell subsets with TRBC1 or TRBC2-restriction. Overall, 20 immunophenotypically distinct TRBC-restricted subsets were identified on 5 (17%) donors and 12 (12%) patients with no T-cell malignancy (Fig. 4F). The presence of T-CUS was associated with advanced age in our cohort (71 vs. 59 years old, *p* = 0.035). The features of these small subsets were similar to those previously described for T-cell clones of uncertain significance [17], including mostly CD8^+^ phenotype (72%), lack of overt tumor-specific immunophenotypic abnormalities, and much smaller median clone size than malignant T-cell subsets (median: 4.7 vs. 69% of lymphocytes, *p* < 0.0001, Fig. 5B).

### Dual TRBC1/2 staining improves clonality assessment and detection of targetable isoforms, compared to TRBC1 staining only

**Fig. 6:**
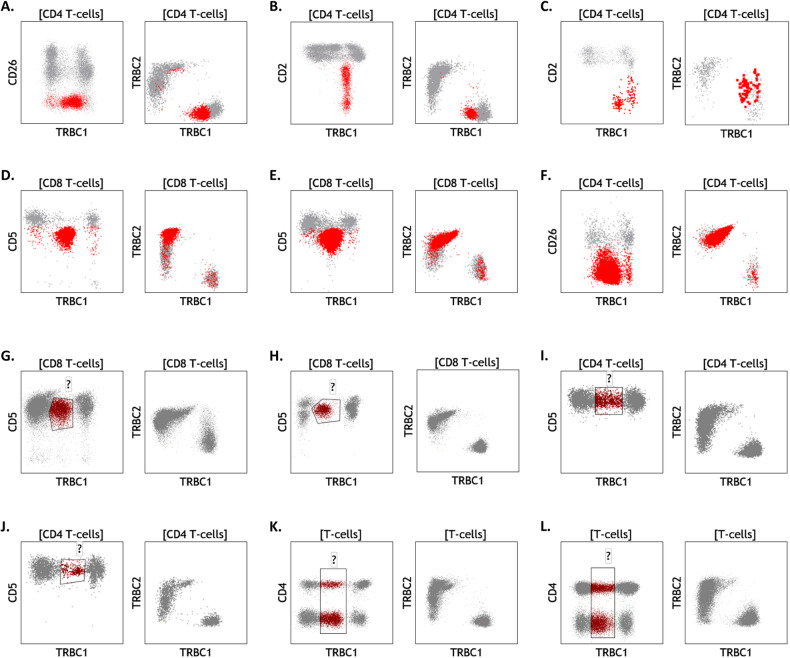
Dual T-cell receptor constant β chain (TRBC)1/TRBC2 assessment resolves common artifacts encountered with TRBC1-only staining. Specimens involved by cutaneous T-cell lymphoma (**A**–**C** and **F**.), T-cell large granular lymphocytic leukemia (**D**, **E**), and samples from patients with no evidence of T-cell malignancy (**G**–**L**), were studied using TRBC1-only staining (left) or dual TRBC1/TRBC2 staining (right). Neoplastic cells (red) with spurious dim expression using TRBC1-only staining are clearly resolved as TRBC1-restricted (**A**–**C**) or TRBC2-restricted (**D**–**F**) neoplasms using dual TRBC1/TRBC2 staining. On benign samples, spurious dim-expressing subsets (maroon) detected with TRBC1-only staining on CD8^+^ T-cells (**G**, **H**), CD4^+^ T-cells (**I**, **J**), or both (**K**, **L**), are completely resolved with dual TRBC1/TRBC2 staining.

Our proposed dual TRBC1 and TRBC2 staining strategy is built upon our extensive experience using TRBC1-only staining in clinical diagnostics [17–22, 24, 25], in addition to similar reports from a few other laboratories [26–28]. To evaluate the added value of dual TRBC1/2 staining, we compared our experimental results with our previously validated diagnostic panels using TRBC1 staining only on the same specimens. Single staining for TRBC1 only produced spurious TRBC1-dim subsets in 24 of 90 (27%) clinical specimens studied with our clinical T-cell immunophenotyping panels (excluding T-ALL cases and specimens with benign T-cell precursors where CD3-dim/TRBC-dim T-cells are expected) (Fig. 6). On these specimens (13 mature T-cell neoplasms and 11 controls), a quantitative estimate of percent TRBC1^+^ events could not be accurately calculated, requiring expert qualitative identification of a monophasic TRBC1 staining pattern to infer clonality (as previously described [20]). This limitation was completely resolved using dual TRBC1/TRBC2 staining, where a clear separation between TRBC1^+^ and TRBC2^+^ events allowed for a straightforward assessment of clonality based on simple TRBC2:TRBC1 ratios (Fig. 6). Importantly, 9 of the 13 mature T-cell neoplasms with dim TRBC1 expression (69%) were shown to be TRBC2-restricted on dual staining (Fig. 6D, E). In addition, 5 of 38 (13%) mature T-cell neoplasms interpreted as TRBC1-negative on single staining (presumed TRBC2-restricted), were actually negative for both TRBC1 and TRBC2 using dual surface and intracellular staining (Fig. 4E). Thus, dual TRBC1/2 staining not only simplifies the analysis of T-cell clonality compared to TRBC1-staining only, but also provides a more reliable determination of the targetable TRBC isoform.

## Discussion

We hereby introduce a novel strategy to identify TCRαβ T-cell neoplasms by flow cytometry, based on the restricted expression of TRBC1 or TRBC2. This approach resembles the routine and broadly utilized assessment of kappa and lambda immunoglobulin light chain restriction for identification of B-cell malignancies [4] (Fig. 1B). It also relies on expert manual segregation (gating) of T-cell subsets exhibiting distinct immunophenotypic features or increased relative abundance concerning for a neoplastic process (Fig. 3A–D), currently the standard of care in clinical flow cytometric analysis of T-cells [1, 2, 29–31]. Rapid confirmation of TRBC-restriction on gated atypical T-cell subsets using TRBC1 and TRBC2 stains within the same panel greatly assists the interpretation of flow cytometry results, obviating the need for a separate T-cell clonality testing in most settings. Given the relative ease by which this novel strategy can be implemented in routine clinical practice, the significant added value to the interpretation of immunophenotypically suspicious T-cell subsets and the potential cost savings, we believe that dual TRBC1/TRBC2 staining is likely to be adopted as a new laboratory standard in the clinical flow cytometric evaluation of leukemic T-cell neoplasms.

Some laboratories, including ours, have already implemented TRBC1 staining only for the assessment of T-cell clonality in clinically validated flow cytometry panels. While we have previously reported the utility of this approach [18–20, 22, 25], the high prevalence of TRBC1-dim subsets (27% in our cohort) complicates the simple calculation of percentage TRBC1-positivity, requiring expert qualitative assessment of TRBC1 staining patterns to infer clonality. Moreover, we show that dual TRBC1/2 staining is needed for the accurate determination of TRBC isoform expression in the context of emerging TRBC1/2-targetted therapies, as 69% of T-cell neoplasms incorrectly interpreted as TRBC1-dim on single staining were shown to be TRBC2-restricted on dual TRBC1/2 staining (Fig. 6D–F). The limitations and staining artifacts observed with TRBC1 staining are reminiscent of our historical experience using only kappa or only lambda immunoglobulin light chain staining with 3 to 4 color legacy flow cytometry panels, a practice that is now obsolete. As such, we anticipate that dual assessment of TRBC1 and TRBC2 expression on two-dimensional plots (as routinely performed for kappa and lambda immunoglobulin light chains) will be strongly favored, effectively overcoming common technical artifacts and providing an accurate determination of the targetable TRBC isoform expressed by T-cell malignancies.

One of the caveats of testing for TRBC-restricted T-cell subsets by flow cytometry is the detection of common small T-CUS in a minority of patients without T-cell neoplasia, including healthy donors (Figs. 4F, 5B). We have previously studied this phenomenon using our current diagnostic T-cell panel with TRBC1-only staining, and shown concurrent TCR-Vβ restriction and an immunophenotypic spectrum closely resembling that of T-LGLL [17]. These findings are well in line with a breadth of literature describing the high prevalence of small clonal T-cell subsets, predominantly in the CD8^+^ T-cell compartment, which are believed to represent physiologic expansions of mostly effector/memory cytotoxic T-cells in response to EBV, CMV, resolved acute infections, neoplastic processes or other sources of antigen exposure [32–39]. Interestingly, a recent deep sequencing study showed the presence of low-frequency somatic mutations on peripheral blood T-cells from 10 of 21 healthy donors (48%), almost exclusively in the CD8-positive T-cell compartment [40], and similar to previous findings on patients with autoimmunity and no T-cell malignancy [41, 42]. Further studies using emerging single-cell sequencing technologies will be needed to determine if T-CUS can be genetically driven by acquired somatic mutations.

The high prevalence of small T-cell clones in the absence of T-cell neoplasia has long complicated the interpretation of ancillary T-cell clonality assays in the diagnosis of T-cell malignancies. In contrast to commonly utilized molecular assays of T-cell clonality, flow cytometry with dual TRBC1 and TRBC2 staining provides useful information regarding the immunophenotype of the T-cell clone detected and its size relative to other T-cell or leukocyte subsets. These are valuable characteristics to establish or suggest a distinction between T-CUS and low-level involvement by a T-cell lymphoproliferative disorder based on expert interpretation and clinical correlation [17]. Moreover, the ability to rapidly and routinely identify T-CUS using dual TRBC1 and TRBC2 stains should facilitate future studies to understand its biology and clinical significance; similar to how other laboratory advances have contributed to our understanding of small clonal B-cell, plasma cell and myeloid proliferation.

In conclusion, we describe a novel and simple strategy to detect clonal TCRαβ T-cells by flow cytometry using dual staining for TRBC1 and TRBC2 within a comprehensive single-tube T-cell panel. This approach can be easily adopted by clinical flow cytometry laboratories for routine analysis, and minimizes the need for a separate T-cell clonality assay. It also provides useful information about the TRBC isoform expressed by T-cell neoplasms for the selection of emerging CAR T-cell therapies targeting TRBC1 or TRBC2 [13, 14]. Our anti-TRBC2 antibody has recently been licensed to selected flow cytometry reagent manufacturers and will soon be commercially available.

### Supplementary information

Supplemental material

## Data Availability

Data sharing is not applicable to this article as no relevant datasets were generated during the current study.

## References

[1] Jevremovic D, Olteanu H (2019). Flow cytometry applications in the diagnosis of T/NK-cell lymphoproliferative disorders. Cytom B Clin Cytom.

[2] Kroft SH, Harrington AM (2022). How I diagnose mature T-cell proliferations by flow cytometry. Am J Clin Pathol.

[3] Tembhare PR, Chatterjee G, Chaturvedi A, Dasgupta N, Khanka T, Verma S (2022). Critical role of flow cytometric immunophenotyping in the diagnosis, subtyping, and staging of T-cell/NK-cell non-Hodgkin’s lymphoma in real-world practice: a study of 232 cases from a tertiary cancer center in India. Front Oncol.

[4] Horna P, Olteanu H, Kroft SH, Harrington AM (2011). Flow cytometric analysis of surface light chain expression patterns in B-cell lymphomas using monoclonal and polyclonal antibodies. Am J Clin Pathol.

[5] Langerak AW, Groenen PJ, Bruggemann M, Beldjord K, Bellan C, Bonello L (2012). EuroClonality/BIOMED-2 guidelines for interpretation and reporting of Ig/TCR clonality testing in suspected lymphoproliferations. Leukemia.

[6] Mahe E, Pugh T, Kamel-Reid S (2018). T cell clonality assessment: past, present and future. J Clin Pathol.

[7] Wang HW, Raffeld M (2019). Molecular assessment of clonality in lymphoid neoplasms. Semin Hematol.

[8] Zhang B, Beck AH, Taube JM, Kohler S, Seo K, Zwerner J (2010). Combined use of PCR-based TCRG and TCRB clonality tests on paraffin-embedded skin tissue in the differential diagnosis of mycosis fungoides and inflammatory dermatoses. J Mol Diagn.

[9] Feng B, Jorgensen JL, Hu Y, Medeiros LJ, Wang SA (2010). TCR-Vbeta flow cytometric analysis of peripheral blood for assessing clonality and disease burden in patients with T cell large granular lymphocyte leukaemia. J Clin Pathol.

[10] Morice WG, Katzmann JA, Pittelkow MR, el-Azhary RA, Gibson LE, Hanson CA (2006). A comparison of morphologic features, flow cytometry, TCR-Vbeta analysis, and TCR-PCR in qualitative and quantitative assessment of peripheral blood involvement by Sezary syndrome. Am J Clin Pathol.

[11] Kotrova M, Novakova M, Oberbeck S, Mayer P, Schrader A, Knecht H (2018). Next-generation amplicon TRB locus sequencing can overcome limitations of flow-cytometric Vbeta expression analysis and confirms clonality in all T-cell prolymphocytic leukemia cases. Cytom A.

[12] Viney JL, Prosser HM, Hewitt CR, Lamb JR, Owen MJ (1992). Generation of monoclonal antibodies against a human T cell receptor beta chain expressed in transgenic mice. Hybridoma.

[13] Maciocia PM, Wawrzyniecka PA, Philip B, Ricciardelli I, Akarca AU, Onuoha SC (2017). Targeting the T cell receptor beta-chain constant region for immunotherapy of T cell malignancies. Nat Med.

[14] Ferrari M, Righi M, Baldan V, Wawrzyniecka P, Bulek A, Kinna A (2024). Structure-guided engineering of immunotherapies targeting TRBC1 and TRBC2 in T cell malignancies. Nat Commun.

[15] Appay V, van Lier RA, Sallusto F, Roederer M (2008). Phenotype and function of human T lymphocyte subsets: consensus and issues. Cytom A.

[16] Horna P, Moscinski LC, Sokol L, Shao H (2019). Naive/memory T-cell phenotypes in leukemic cutaneous T-cell lymphoma: putative cell of origin overlaps disease classification. Cytom B Clin Cytom.

[17] Shi M, Olteanu H, Jevremovic D, He R, Viswanatha D, Corley H (2020). T-cell clones of uncertain significance are highly prevalent and show close resemblance to T-cell large granular lymphocytic leukemia. Implications for laboratory diagnostics. Mod Pathol.

[18] Horna P, Shi M, Jevremovic D, Craig FE, Comfere NI, Olteanu H (2021). Utility of TRBC1 expression in the diagnosis of peripheral blood involvement by cutaneous T-cell lymphoma. J Invest Dermatol.

[19] Berg H, Otteson GE, Corley H, Shi M, Horna P, Jevremovic D (2021). Flow cytometric evaluation of TRBC1 expression in tissue specimens and body fluids is a novel and specific method for assessment of T-cell clonality and diagnosis of T-cell neoplasms. Cytom B Clin Cytom.

[20] Shi M, Jevremovic D, Otteson GE, Timm MM, Olteanu H, Horna P (2020). Single antibody detection of T-cell receptor alphabeta clonality by flow cytometry rapidly identifies mature T-cell neoplasms and monotypic small CD8-positive subsets of uncertain significance. Cytom B Clin Cytom.

[21] Horna P, Otteson G, Shi M, Seheult JN, Jevremovic D, Olteanu H (2022). Improved semiautomated detection of TRBC-restricted Sezary cells unveils a spectrum of clonal cluster immunophenotypes. Blood.

[22] Horna P, Shi M, Olteanu H, Johansson U (2021). Emerging role of T-cell receptor constant beta chain-1 (TRBC1) expression in the flow cytometric diagnosis of T-cell malignancies. Int J Mol Sci.

[23] Mallis RJ, Bai K, Arthanari H, Hussey RE, Handley M, Li Z (2015). Pre-TCR ligand binding impacts thymocyte development before alphabetaTCR expression. Proc Natl Acad Sci USA.

[24] Horna P, Otteson GE, Shi M, Jevremovic D, Yuan J, Olteanu H (2022). Flow cytometric evaluation of surface and cytoplasmic TRBC1 expression in the differential diagnosis of immature T-cell proliferations. Am J Clin Pathol.

[25] Horna P, Olteanu H, Jevremovic D, Otteson GE, Corley H, Ding W (2021). Single-antibody evaluation of T-cell receptor beta constant chain monotypia by flow cytometry facilitates the diagnosis of T-cell large granular lymphocytic leukemia. Am J Clin Pathol.

[26] Novikov ND, Griffin GK, Dudley G, Drew M, Rojas-Rudilla V, Lindeman NI (2019). Utility of a simple and robust flow cytometry assay for rapid clonality testing in mature peripheral T-cell lymphomas. Am J Clin Pathol.

[27] Munoz-Garcia N, Lima M, Villamor N, Moran-Plata FJ, Barrena S, Mateos S (2021). Anti-TRBC1 antibody-based flow cytometric detection of T-cell clonality: standardization of sample preparation and diagnostic implementation. Cancers.

[28] Martin-Moro F, Martin-Rubio I, Garcia-Vela JA (2022). TRBC1 expression assessed by flow cytometry as a novel marker of clonality in cutaneous alphabeta T-cell lymphomas with peripheral blood involvement. Br J Dermatol.

[29] Jamal S, Picker LJ, Aquino DB, McKenna RW, Dawson DB, Kroft SH (2001). Immunophenotypic analysis of peripheral T-cell neoplasms. A multiparameter flow cytometric approach. Am J Clin Pathol.

[30] Horna P, Wang SA, Wolniak KL, Psarra K, Almeida J, Illingworth AJ (2021). Flow cytometric evaluation of peripheral blood for suspected Sezary syndrome or mycosis fungoides: international guidelines for assay characteristics. Cytom B Clin Cytom.

[31] Horna P, Deaver DM, Qin D, Moscinski LC, Sotomayor EM, Glass LF (2014). Quantitative flow cytometric identification of aberrant T cell clusters in erythrodermic cutaneous T cell lymphoma. Implications for staging and prognosis. J Clin Pathol.

[32] Maini MK, Gudgeon N, Wedderburn LR, Rickinson AB, Beverley PC (2000). Clonal expansions in acute EBV infection are detectable in the CD8 and not the CD4 subset and persist with a variable CD45 phenotype. J Immunol.

[33] Blackman MA, Woodland DL (2011). The narrowing of the CD8 T cell repertoire in old age. Curr Opin Immunol.

[34] Hadrup SR, Strindhall J, Kollgaard T, Seremet T, Johansson B, Pawelec G (2006). Longitudinal studies of clonally expanded CD8 T cells reveal a repertoire shrinkage predicting mortality and an increased number of dysfunctional cytomegalovirus-specific T cells in the very elderly. J Immunol.

[35] Khan N, Shariff N, Cobbold M, Bruton R, Ainsworth JA, Sinclair AJ (2002). Cytomegalovirus seropositivity drives the CD8 T cell repertoire toward greater clonality in healthy elderly individuals. J Immunol.

[36] Fairfax BP, Taylor CA, Watson RA, Nassiri I, Danielli S, Fang H (2020). Peripheral CD8(+) T cell characteristics associated with durable responses to immune checkpoint blockade in patients with metastatic melanoma. Nat Med.

[37] Singleton TP, Yin B, Teferra A, Mao JZ (2015). Spectrum of clonal large granular lymphocytes (LGLs) of alphabeta T cells: T-cell clones of undetermined significance, T-cell LGL leukemias, and T-cell immunoclones. Am J Clin Pathol.

[38] Bernardin F, Doukhan L, Longone-Miller A, Champagne P, Sekaly R, Delwart E (2003). Estimate of the total number of CD8+ clonal expansions in healthy adults using a new DNA heteroduplex-tracking assay for CDR3 repertoire analysis. J Immunol Methods.

[39] Ely KH, Ahmed M, Kohlmeier JE, Roberts AD, Wittmer ST, Blackman MA (2007). Antigen-specific CD8+ T cell clonal expansions develop from memory T cell pools established by acute respiratory virus infections. J Immunol.

[40] Savola P, Martelius T, Kankainen M, Huuhtanen J, Lundgren S, Koski Y (2020). Somatic mutations and T-cell clonality in patients with immunodeficiency. Haematologica.

[41] Valori M, Jansson L, Kiviharju A, Ellonen P, Rajala H, Awad SA (2017). A novel class of somatic mutations in blood detected preferentially in CD8+ cells. Clin Immunol.

[42] Savola P, Kelkka T, Rajala HL, Kuuliala A, Kuuliala K, Eldfors S (2017). Somatic mutations in clonally expanded cytotoxic T lymphocytes in patients with newly diagnosed rheumatoid arthritis. Nat Commun.

